# Evaluation of the Predictive Potential of 18F-FDG PET and DWI Data Sets for Relevant Prognostic Parameters of Primary Soft-Tissue Sarcomas

**DOI:** 10.3390/cancers13112753

**Published:** 2021-06-01

**Authors:** Michal Chodyla, Aydin Demircioglu, Benedikt M. Schaarschmidt, Stefanie Bertram, Janna Morawitz, Sebastian Bauer, Lars Podleska, Christoph Rischpler, Michael Forsting, Ken Herrmann, Lale Umutlu, Johannes Grueneisen

**Affiliations:** 1 Department of Diagnostic and Interventional Radiology, University Hospital Essen, University of Duisburg-Essen, 45147 Essen, Germany; Michal-Kamil.Chodyla@uk-essen.de (M.C.); aydin.demircioglu@uk-essen.de (A.D.); Benedikt.Schaarschmidt@uk-essen.de (B.M.S.); michael.forsting@uk-essen.de (M.F.); lale.umutlu@uk-essen.de (L.U.); 2 Institute of Pathology, University Hospital Essen, University of Duisburg-Essen, 45147 Essen, Germany; stefanie.bertram@uk-essen.de; 3 Department of Diagnostic and Interventional Radiology, University Hospital Dusseldorf, University of Dusseldorf, 40225 Dusseldorf, Germany; Janna.Morawitz@med.uni-duesseldorf.de; 4 Sarcoma Center, Western German Cancer Center, University of Duisburg-Essen, 45147 Essen, Germany; Sebastian.Bauer@uk-essen.de; 5 Sarcoma Surgery Division, Department of General, Visceral and Transplantation Surgery, University Hospital Essen, University of Duisburg-Essen, 45147 Essen, Germany; Lars.podleska@uk-essen.de; 6 Department of Nuclear Medicine, University Hospital Essen, University of Duisburg-Essen, 45147 Essen, Germany; christoph.rischpler@uk-essen.de (C.R.); ken.herrmann@uk-essen.de (K.H.)

**Keywords:** soft-tissue sarcoma, 18F-FDG PET, MRI, isolated limb perfusion

## Abstract

**Simple Summary:**

In this study, we assessed the potential of simultaneously acquired 18F-fluorodeoxyglucose positron emission tomography (18F-FDG PET) and magnetic resonance imaging-derived quantitative imaging parameters to predict the tumor grade, metastatic status, and response to neoadjuvant therapy of primary soft-tissue sarcomas of the extremities. Based on the results of the present study, quantifications of the 18F-FDG PET uptake provide useful prognostic data for the evaluation of histopathological response to neoadjuvant treatment as well as the aggressiveness of high-risk sarcomas, whereas no correlation between the different outcome variables and the results for tumor size and diffusion-weighted imaging-derived apparent diffusion coefficient values was found. Accordingly, measurements of the metabolic activity of primary and untreated soft-tissue sarcomas could non-invasively deliver relevant information, that may be used for treatment planning and risk-stratification of sarcoma patients in a pretherapeutic setting.

**Abstract:**

Background: To evaluate the potential of simultaneously acquired 18F-FDG PET- and MR-derived quantitative imaging data sets of primary soft-tissue sarcomas for the prediction of neoadjuvant treatment response, the metastatic status and tumor grade. Methods: A total of 52 patients with a high-risk soft-tissue sarcoma underwent a 18F-FDG PET/MR examination within one week before the start of neoadjuvant treatment. For each patient, the maximum tumor size, metabolic activity (SUVs), and diffusion-restriction (ADC values) of the tumor manifestations were determined. A Mann–Whitney-U test was used, and ROC analysis was performed to evaluate the potential to predict histopathological treatment response, the metastatic status or tumor grade. The results from the histopathological analysis served as reference standard. Results: Soft-tissue sarcomas with a histopathological treatment response revealed a significantly higher metabolic activity than tumors in the non-responder group. In addition, grade 3 tumors showed a significant higher 18F-FDG uptake than grade 2 tumors. Furthermore, no significant correlation between the different outcome variables and tumor size or calculated ADC-values could be identified. Conclusion: Measurements of the metabolic activity of primary and untreated soft-tissue sarcomas could non-invasively deliver relevant information that may be used for treatment planning and risk-stratification of high-risk sarcoma patients in a pretherapeutic setting.

## 1. Introduction

Soft-tissue sarcomas represent a heterogeneous group of malignant tumors that develop from mesenchymal tissues and account for approximately 1% of solid cancers in adults worldwide [1]. Highly accurate initial diagnostics of these tumors are of significant importance to identify the most appropriate and frequently multimodal treatment strategy. Moreover, a specific local and whole-body staging histopathological verification of the primary tumor is mandatory, since different sarcoma subtypes and tumor grades necessitate partially specific therapeutic interventions [2,3,4]. In addition, the integration of these data provides useful information to assess patients’ prognosis. As a standard method, histopathologic verification after open or core needle biopsy is performed on specimens from representative parts of the partially large tumor manifestations [5,6]. However, this procedure does not allow for a histological workup of the entire tumor lesion; thus, possibly relevant information about the underlying tumor biology may be not identified at the time of initial diagnosis. In this context, imaging has become an evolving role for primary diagnostics and could deliver relevant complementary data for the evaluation and risk stratification of primary soft-tissue masses [7,8]. In addition to investigations of the local extent and specific tissue characteristics of these tumors, several functional and quantitative imaging parameters have been put into the focus of previous studies, for example, to evaluate their potential to determine treatment response or to predict patients’ outcome [9,10,11]. With the implementation of integrated positron emission tomography (PET)/magnetic resonance imaging (MRI) systems, certain morphological, functional, and metabolic data sets can be obtained within one examination. A few studies already reported that both, 18F-fluorodeoxyglucose (18F-FDG) PET as well as MRI-derived diffusion-weighted imaging (DWI) data sets can be applied for response evaluation of soft-tissue sarcomas, based on measurable changes of these quantitative parameters under treatment [12,13,14,15]. In contrast, tumor size measurements have been shown to be insufficient for reliable therapy response assessment, yet it is well known that the initial extent of soft-tissue sarcomas has a considerable prognostic relevance [9,16,17]. Therefore, this study aimed to evaluate and compare the potential of certain clinically established 18F-FDG PET- and DWI-derived quantitative imaging parameters, simultaneously obtained from primary and treatment-naive high-risk soft-tissue sarcomas, to predict therapy response to neoadjuvant isolated limb perfusion (ILP), the tumor grade, and the metastatic status.

## 2. Material and Methods

### 2.1. Patients

The study was conducted according to the guidelines of the Declaration of Helsinki and was approved by the institutional review board. Written informed consent was obtained from each patient before the examination. A total of 52 patients (mean age 53.9 ± 16.4 years) with a primary and untreated high-grade soft-tissue sarcoma of the extremities were included in this prospective study and underwent a 18F-FDG PET/MR examination within one week before neoadjuvant ILP. This surgical intervention was performed under general anesthesia and mild hyperthermia of 39° via a brachial/axillar approach for the treatment of tumors of the arms or a femoral/axillar approach for tumors of the legs. First, recombinant human tumor necrosis factor-α (TNF-α, Beromun, Boehringer–Ingelheim, Germany) was applied, adjusted to 0.25 mg/L perfused tissue volume and with a delay of 15 min melphalan (L-phenylalanine mustard) was administered at a concentration of 11 mg/L for tumors of the lower limb and 13 mg/L for sarcoma manifestations of the upper limb. With a mean delay of 46.5 ± 8.8 days after this neoadjuvant therapeutic procedure, all tumors were surgically resected, and histopathological analysis was performed for the determination of treatment response or non-response.

### 2.2. PET/MRI

All examinations were obtained with an integrated PET/MR system (Biograph mMR, Siemens Healthineers, Germany) and a delay of 60 min after intravenous injection of a weight-adapted dosage of 18F-FDG. The 18F-FDG PET- and MR-data sets were acquired simultaneously using 1–2 bed positions covering the entire tumor manifestation and with a PET-data acquisition time of 10 min per bed. Subsequently, PET images were reconstructed using the iterative ordered-subset expectation maximization algorithm, 3 iterations and 21 subsets, a Gaussian filter with 4 mm full-width at half maximum, and a 344 × 344 image matrix. Attenuation correction of PET images was performed automatically on a four-compartment model attenuation map (μ-map) calculated from fat-only and water-only data sets obtained by Dixon-based sequences, facilitating a segmentation into background, lung, fat, and soft-tissue. The MR imaging data sets were acquired using dedicated mMR RF body-phased array coils and spine coils. Table 1 provides detailed information about the MR imaging protocol and sequence parameters.

### 2.3. Image Analysis

The PET/MR imaging data sets were analyzed by two experienced physicians (B.M.S., J.G.) in consensus, using a viewing software for hybrid imaging (Syngo.via B30; Siemens Healthineers, Germany). The readers were blinded regarding the results of histopathological analysis (tumor grade and regression grade after surgical tumor resection) as well as the metastatic status of the patients but were informed about the diagnosis of a primary soft-tissue sarcoma of the extremity and the neoadjuvant treatment procedure.

First, the maximum tumor diameter was measured on contrast-enhanced fat-saturated T1w TSE MR images. In a second step, diffusion-restriction of the primary tumor manifestations was quantified on the ADC-map that was generated automatically by the scanner software (Syngo MR B18P, Siemens Healthineers, Germany) using three different b-values (b = 0 s/mm^2^, b = 500 s/mm^2^, b = 1000 s/mm^2^). Therefore, the soft-tissue tumors were identified on diffusion-weighted images, followed by a manually placement of a polygonal region of interest (ROI) on every slice of the corresponding ADCmap, encompassing the entire tumor lesion. Subsequently, the ADC values (ADCmin and ADCmean) were automatically calculated by the viewing software. Furthermore, for measurements of the metabolic activity of the tumor manifestations a volume of interest was manually placed on fused 18F-FDG PET/MR images and the standardized uptake volumes (SUVmax and SUVmean) were automatically generated.

### 2.4. Reference Standard

Histopathological analyses were performed by an experienced soft-tissue sarcoma pathologist. Tumor grades (grade 2 or grade 3) were determined based on histopathological analysis of initial biopsy specimens and in accordance with the histological grading system of the French Federation of Cancer Centers Sarcoma Group (FNCLCC) [18]. For the determination of treatment response/non-response of the primary soft-tissue sarcomas to neoadjuvant ILP, histopathological results after surgical resection of the tumors were used. Treatment effects were assessed according to the grading scheme by Salzer-Kuntschik [19], initially introduced for grading the amount of histological tumor regression of osteosarcomas under chemotherapy, which has also been shown valuable to classify therapy-induced histological findings of soft-tissue sarcomas [20]. Therefore, histopathological results were divided into six different regression grades (grade 1 = no vital tumor, grade 2 = single vital tumor cell or one cluster/5 mm, grade 3 < 10% vital tumor, grade 4 = 10–50% vital tumor, grade 5 > 50% vital tumor, and grade 6 = no effect of therapy) based on the percentage of the viable tumor amount of the surgical specimen. In concordance with previous publications, tumors with a regression grade 1–3 were defined as histopathological responders and a grade 4–6 as non-responders [17,21]. Furthermore, for the determination of the metastatic status at the time of initial diagnosis/before the start of neoadjuvant treatment, the results from initial tumor staging (chest and abdominal computed tomography (CT), local and whole-body PET/MRI scan) as well as follow-up imaging (chest and abdominal CT or 18F-FDG PET/CT and locoregional MRI), and histopathological sampling (e.g., lymphadenectomy, lung nodule resection or biopsy) were taken into account.

### 2.5. Statistical Analysis

Statistical analysis was performed using the R software environment for statistical computing and graphics (version 3.6.3). Quantitative PET/MR-derived imaging parameters in dependence of patients’ histopathological treatment response, metastatic status, and tumor grade are presented as mean values ± standard deviation (SD). A Mann–Whitney-U test was applied to test for significant differences of the results. *p*-Values < 0.05 were considered to be statistically significant. In addition, a receiver operating characteristic (ROC) analysis was performed to determine the diagnostic ability of the different quantitative imaging parameters to predict treatment response under ILP, the metastatic status and tumor grade (grade 2 vs. grade 3). Corresponding area-under-the-curve (AUC) values were computed and statistically compared using a bootstrap test with 2000 replicates.

## 3. Results

### 3.1. Patients

**Figure 1:**
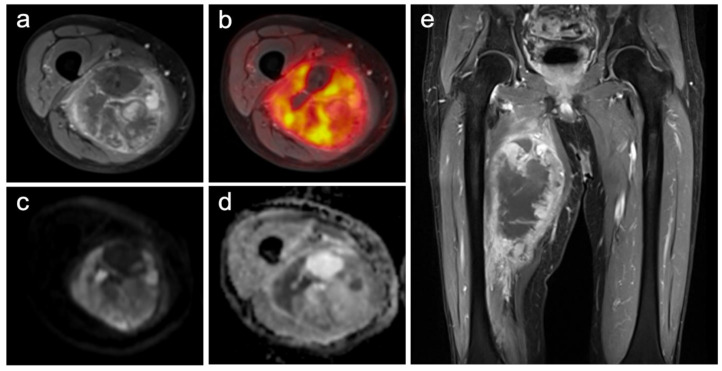
Images of a primary soft-tissue sarcoma (undifferentiated pleomorphic sarcoma) of the right upper leg ((**a**) contrast-enhanced T1w TSE with fat-sat axial; (**b**) PET/MRI; (**c**) DWI b-1000; (**d**) ADC-map; (**e**) contrast-enhanced T1w TSE with fat-sat coronal). The large and partially necrotic tumor manifestation (23 mm) shows a pathologic glucose metabolism (SUVmax: 15.1; SUVmean: 7.5) and parts of restricted diffusion (ADCmean 1491; ADCmin 487). The reference standard revealed a grade 3 tumor with a good histopathological treatment response (regression grade 2), and metastatic spread could be identified.

All 52 patients with a primary high-risk soft-tissue sarcoma of the extremities successfully completed the pretherapeutic PET/MR examination without any relevant side effects (Figure 1). Among them, 18/52 (35%) patients revealed a grade 2 soft-tissue sarcoma and 34/52 (65%) patients a grade 3 tumor manifestation, in accordance with the histological grading system of the FNCLCC. Furthermore, in 14/52 (27%) patients, lymph node and/or distant metastases were present at the time of initial diagnosis, whereas in the remaining 38/52 (73%) patients, no metastatic spread could be identified. In addition, histopathological analysis after neoadjuvant ILP and subsequent surgical tumor resection categorized 28/52 (54%) patients as therapy responders (regression grades 1–3) and 24/52 (46%) patients as non-responders (regression grades 4–6). The different soft-tissue sarcoma subtypes of all 52 patients are shown in Table 2.

### 3.2. Quantitative Image Analysis

**Figure 2:**
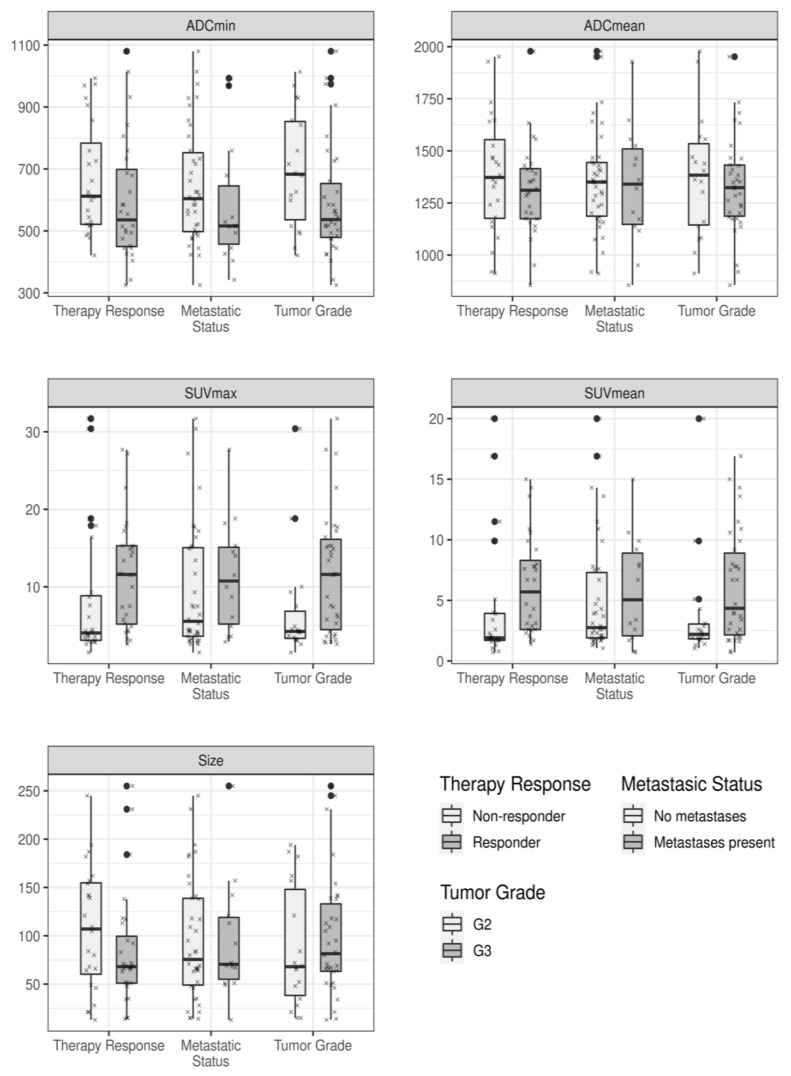
Measured quantitative imaging parameters in dependence of the different outcome variables, displayed as boxplots. Each box indicates the first and third quartile, while the whiskers indicate the 1.5 * inter-quartile range. Outliers, defined as values outside this range, are indicated by bold black dots. In addition, all measured values are indicated by small crosses.

The calculated mean values of the different 18F-FDG PET/MRI-derived quantitative imaging parameters, in dependence of histopathological treatment response, the metastatic status, and tumor grade are displayed in Table 3 and Figure 2. Patients with histopathologically proven treatment response showed a significantly higher pretherapeutic SUVmax (*p*-value: 0.020) and SUVmean (*p*-value: 0.007), when compared to those patients of the non-responder group (Table 4). In addition, soft-tissues sarcomas of the extremities with a tumor grade 3 revealed a significantly higher SUVmax (*p*-value: 0.024) and SUVmean (*p*-value: 0.036) than grade 2 tumors. Table 5 provides an overview of the distribution of therapy responders and non-responders in dependence of the tumor grade. In contrast, the differences among these metabolic imaging parameters (SUVs) between primary tumor manifestations of patients with or without metastatic spread were not significant.

Moreover, primary soft-tissue sarcomas with grade 3 showed a lower ADCmin value in comparison to grade 2 tumors, but the differences between the values were not statistically significant (*p*-value: 0.053). The differences in the calculated mean values for tumor size and the ADCmean, considering patients’ treatment response, metastatic status, and tumor grade were not significant.

**Figure 3:**
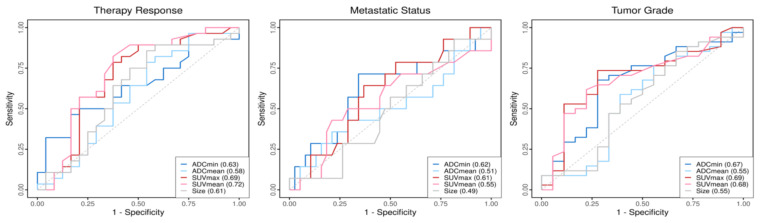
ROCs of the quantitative imaging parameters in dependence of the different outcome variables. Corresponding AUC values of the parameters are shown within the boxes.

The results of ROC analysis are displayed in Figure 3. For the prediction of the histopathological treatment response to neoadjuvant ILP, the SUVmean revealed the highest AUC value among the different quantitative imaging variables. Furthermore, the SUVmax showed the highest AUC value to discriminate between soft-tissue sarcomas with grade 3 or grade 2.

## 4. Discussion

Highly accurate initial diagnostics of patients with a diagnosis of a primary soft-tissue sarcoma manifestation is of significant importance to initiate the most appropriate treatment strategy and to receive important information about a patients’ prognosis. A few studies have already demonstrated the potential of integrated PET/MR imaging for local and whole-body staging as well as treatment monitoring of sarcoma patients [14,22,23]. Furthermore, this new hybrid imaging modality enables simultaneous acquisition of certain quantitative imaging parameters, reflecting different aspects of the underlying tumor biology [24,25]. It has previously been reported that histopathological response can be predicted based on changes in the metabolic activity of the tumors under neoadjuvant treatment. Benz et al. demonstrated that a reduction in tumor 18F-FDG uptake of more than 35% after the first cycle of neoadjuvant chemotherapy is a sensitive predictor for therapy response [26]. Another article by Evelevitch et al. described that a 60% decrease in tumor 18F-FDG uptake, had a 100% sensitivity and a 71% specificity for histopathological response under neoadjuvant treatment [27]. While the majority of studies investigated the treatment effects of intravenous chemotherapy, our patient cohort consisted of patients that underwent neoadjuvant ILP before resection of the primary tumors. This invasive treatment procedure is most frequently applied in western European countries, especially for the treatment of locally advanced and primarily irresectable extremity soft-tissue sarcomas to achieve local tumor control and limb salvage. In a recently published work, the authors showed that therapy response of soft-tissue sarcomas under neoadjuvant ILP was predictable due to the significant changes in both the tumors’ 18F-FDG uptake and diffusion–restriction [10]. In the present study, solely primary and untreated tumors were evaluated prior to ILP. In this patient cohort, sarcoma manifestations with a good histopathological response had a significantly higher metabolic activity, when compared to the non-responder group. The phenomenon that metabolically more active and potentially more aggressive tumors show higher response rates may be explained by the tumor–biological changes induced by hyperthermic ILP. While melphalan is considered the main drug, its direct cytotoxic influence is supplemented by recombinant human TNF-α. As an early effect, TNF-α induces an increased uptake of the simultaneously administered alkylating agent into the tumor under ILP, accompanied with its important antiangiogenetic effects, leading to delayed selective destruction of tumor-associated vessels and subsequent tumor regression. [28,29]. Some multicenter trials reported that the combined administration of these two agents led to significantly higher response rates and limb salvage rates with this neoadjuvant treatment procedure [30,31,32]. Moreover, it is generally assumed that due to the additive use of TNF-α, strongly vascularized and, thus, frequently more aggressive tumors respond better to this neoadjuvant treatment procedure [20].

In this context, several previously mentioned articles described that reliable prediction of treatment response could be achieved by the determination of measurable changes in the metabolic activity between pretherapeutic 18F-FDG PET scans and further examinations under or at the end of neoadjuvant therapy. However, our data underline, specifically, the usefulness of FDG-PET data quantifications of the primary and therapy-naive soft-tissue sarcomas. This step could non-invasively deliver valuable information to identify patients that might be more suitable to undergo neoadjuvant ILP. Furthermore, it would be of interest, to investigate, whether these results can also be observed in patient cohorts undergoing neoadjuvant systemic treatment.

In addition, our results reveal that grade 3 tumors have a significantly higher initial 18F-FDG uptake than soft-tissue sarcomas with tumor grade 2. This finding is in line with previous observations, that the metabolic activity closely correlates with tumors’ aggressiveness, which has been demonstrated for several different malignancies including sarcomas [33,34]. Eary and colleagues reported in a previous publicatione that the 18F-FDG uptake of sarcomas differed significantly between low-, intermediate-, and high-grade tumors using the NCI grading system [35]. Another meta-analysis, including 29 studies and a total of 1163 patients with bone or soft-tissue sarcomas, demonstrated that the SUVmean can be used to reliably discriminate between low- and high-grade sarcomas [36]. Although grade 2 and 3 soft-tissue sarcomas are frequently summarized as high-grade tumors, patients show considerable differences in survival [37,38]. Focusing on the prognostic impact of this quantitative imaging parameter, Herrmann et al. showed that 18F-FDG-PET data allow survival prediction already after the initial cycle of neoadjuvant chemotherapy in patients with STS [9]. Fendler et al. reported that the evaluation of the metabolic activity enables prediction of progression-free survival as well time to local and distant disease progression of STS under neoadjuvant treatment [39]. In addition, Kubo and colleagues demonstrated in a meta-analysis comprising 514 sarcoma patients that a high pre-treatment SUVmax predicts a significantly shorter overall survival period than a low SUVmax [40].

Moreover, the initial size of the tumors at the time of diagnosis has been shown to have a significant effect on patients’ prognosis [16]. However, we could not find any significant correlation between the size of the primary tumors and the outcome variables evaluated in this study. Putting the focus on functional quantitative MR imaging, some articles have already investigated the potential of MR/DWI-derived ADC values for treatment response prediction of soft-tissue sarcomas. The authors were able to discriminate between therapy responders and non-responders due to the significant differences in measurable changes in these quantitative imaging parameters under neoadjuvant treatment [12,41]. In our cohort, we did not obtain any relevant predictive data from the ADC values for a reliable differentiation between histopathological responders and non-responders as well as for the identification of patients with metastatic spread, solely analyzing the quantitative information about the tumor structure pre-therapeutically. Only the ADCmin, reflecting the spot of the highest cellularity within the primary tumors, revealed the tendency to be lower in grade 3 than in grade 2 tumors, yet the differences almost reached the significance level (*p*-value: 0.053). Chhabra and colleagues already described in a previous publication that DWI data can be helpful in grading soft-tissue malignancies. In their work, measured ADC values significantly decreased with increasing grades of primary tumors [42]. In another study by Robba and colleagues, the authors could successfully apply ADC values to differentiate benign from malignant soft-tissue tumors as well as to discriminate between grade 2 and 3 tumors [43]. Since this topic has been investigated in only a few studies with generally only a limited number of cases so far, it should be focused on more extensively in future works. This observation also reveals the major limitation of the present publication. The number of patients included in this study was relatively small; thus, our findings should be considered preliminary and need to be confirmed in further studies comprising larger patient cohorts. In addition, we included patients with several soft-tissue sarcoma subtypes, potentially exhibiting a different tumor biological behavior, which presumably had an influence on the determined quantitative imaging parameters and consequently our results. Then, imaging data were obtained between May 2014 and September 2019; hence, no reliable follow-up period of the patients was available to investigate potential correlations between the quantitative imaging variables and survival data.

## 5. Conclusions

Quantifications of the 18F-FDG PET uptake of primary and therapy-naive soft-tissue sarcomas, provide useful prognostic data for the determination of histopathological treatment response to neoadjuvant ILP as well as the aggressiveness of high-risk sarcomas, whereas no significant correlation between the different outcome variables and the initial tumor size and DWI-derived ADC values was found. Accordingly, measurements of the initial metabolic activity of soft-tissue sarcomas could non-invasively deliver relevant information that could be used for treatment planning and risk stratification of high-risk sarcoma patients in a pretherapeutic setting.

## Figures and Tables

**Table 1:** MR imaging protocol and sequence parameters.

Sequence Parameters	T1w VIBE Dixon (Coronal)	STIR (Coronal)	T1w TSE (Axial)	T2w TSE (Axial)	DW EPI (b-values: 0, 500, 1000 s/mm^2^; Axial)	T1w VIBE Dynamic (Axial)	T1w TSE Post Contrast (Axial)	T1w TSE Post Contrast (Axial)
Slices (mm)	3.12	5	5	5	5	3.5	5	5
Repetition time (ms)	3.6	5990	616	4860	7400	4.32	542	663
Echo time (ms)	1st: 1.23; 2nd 2.46	57	12	106	72	2.21	13	13
Field of view (mm)	500	380	380	380	420	380	380	380
Matrix size	192 × 79	384 × 288	512 × 256	512 × 256	160 × 120	512 × 308	512 × 256	512 × 256

**Table 2:** Distribution of histological subtypes of soft-tissue sarcomas for all patients included in this study.

Histological Subtype	Number of Patients
Undifferentiated pleomorphic sarcoma	16
Synovial sarcoma	9
Liposarcoma	8
Undifferentiated spindle cell sarcoma	5
Leiomyosarcoma	5
Myxofibrosarcoma	3
Epithelioid sarcoma	2
Fibrosarcoma	2
Clear cell sarcoma	1
Malignant peripheral nerve sheath tumor	1
Total	52

**Table 3:** Results of quantitative image analysis (mean values +/− standard deviation) of the 18F-FDG PET/MRI-derived quantitative imaging parameters, in dependence of histopathological treatment response, metastatic status and tumor grade.

Category		Size (mm)	SUVmax	SUVmean	ADCmin	ADCmean
Therapy response	Responder (*n* = 28)	83.9 +/− 58.8	11.6 +/− 7.1	6.2 +/− 4.1	596.5 +/− 197.0	1315.2 +/− 220.4
	Non-responder (*n* = 24)	107.4 +/− 63.7	8.2 +/− 8.6	4.2 +/− 5.1	667.0 +/− 180.8	1385.4 +/− 284.8
Metastatic status	No metastases (*n* = 38)	94.9 +/− 62.7	9.6 +/− 8.2	5.1 +/− 4.8	646.9 +/− 187.6	1352.7 +/− 242.2
	Metastases present (*n* = 14)	94.3 +/− 60.8	11.4 +/− 7.2	5.8 +/− 4.4	580.7 +/− 199.4	1333.9 +/− 286.5
Tumor grade	Grade 2 (*n* = 18)	87.4 +/− 62.9	6.8 +/− 7.1	3.8 +/− 4.5	699.6 +/− 189.7	1379.4 +/− 292.9
	Grade 3 (*n* = 34)	98.6 +/− 61.5	11.7 +/− 7.9	6.1 +/− 4.6	591.7 +/− 183.9	1330.8 +/− 230.5

**Table 4:** Calculated *p*-values for the differences in the quantitative imaging parameters in dependence of the outcome variables.

Category	Size (mm)	SUVmax	SUVmean	ADCmin	ADCmean
Therapy response	0.163	0.020	0.007	0.108	0.317
Metastatic status	0.893	0.252	0.563	0.180	0.893
Tumor grade	0.551	0.024	0.036	0.053	0.570

**Table 5:** Distribution of therapy responders and non-responders in patients with soft-tissue sarcomas grade 2 or grade 3.

Category	Grade 2	Grade 3	Sum
Responder	9	19	28
Non-responder	9	15	24
Sum	18	34	52

## Data Availability

Available upon reasonable request.

## References

[1] Demetri G.D., Antonia S., Benjamin R.S., Bui M.M., Casper E.S., Conrad E.U., De Laney T.F., Ganjoo K.N., Heslin M.J., Hutchinson R.J. (2010). Soft tissue sarcoma. J. Natl. Compr. Canc. Netw..

[2] Casali P.G., Abecassis N., Aro H.T., Bauer S., Biagini R., Bielack S., Bonvalot S., Boukovinas I., Bovee J., Brodowicz T. (2018). Soft tissue and visceral sarcomas: ESMO-EURACAN Clinical Practice Guidelines for diagnosis, treatment and follow-up. Ann. Oncol..

[3] Von Mehren M., Randall R.L., Benjamin R.S., Boles S., Bui M.M., Ganjoo K.N., George S., Gonzalez R.J., Heslin M.J., Kane J.M. (2018). Soft Tissue Sarcoma, Version 2.2018, NCCN Clinical Practice Guidelines in Oncology. J. Natl. Compr. Canc. Netw..

[4] Von Mehren M., Kane J.M., Bui M.M., Choy E., Connelly M., Dry S., Ganjoo K.N., George S., Gonzalez R.J., Heslin M.J. (2020). NCCN Guidelines Insights: Soft Tissue Sarcoma, Version 1.2021. J. Natl. Compr. Canc. Netw..

[5] Grimer R., Judson I., Peake D., Seddon B. (2010). Guidelines for the management of soft tissue sarcomas. Sarcoma.

[6] Grünewald T.G., Alonso M., Avnet S., Banito A., Burdach S., Cidre-Aranaz F., Di Pompo G., Distel M., Dorado-Garcia H., Garcia-Castro J. (2020). Sarcoma treatment in the era of molecular medicine. EMBO Mol. Med..

[7] Clarkson P., Ferguson P.C. (2004). Primary multidisciplinary management of extremity soft tissue sarcomas. Curr. Treat. Options Oncol..

[8] Noebauer-Huhmann I.M., Weber M.A., Lalam R.K., Trattnig S., Bohndorf K., Vanhoenacker F., Tagliafico A., van Rijswijk C., Vilanova J.C., Afonso P.D. (2015). Soft Tissue Tumors in Adults: ESSR-Approved Guidelines for Diagnostic Imaging. Semin. Musculoskelet. Radiol..

[9] Herrmann K., Benz M.R., Czernin J., Allen-Auerbach M.S., Tap W.D., Dry S.M., Schuster T., Eckardt J.J., Phelps M.E., Weber W.A. (2012). 18F-FDG-PET/CT Imaging as an early survival predictor in patients with primary high-grade soft tissue sarcomas undergoing neoadjuvant therapy. Clin. Cancer Res..

[10] Chodyla M., Demircioglu A., Schaarschmidt B.M., Bertram S., Bruckmann N.M., Haferkamp J., Li Y., Bauer S., Podleska L.E., Rischpler C. (2020). Evaluation of (18)F-FDG PET and DWI datasets for the prediction of therapy response of soft tissues sarcomas under neoadjuvant isolated limb perfusion. J. Nucl. Med..

[11] Lee J.H., Yoon Y.C., Seo S.W., Choi Y.L., Kim H.S. (2020). Soft tissue sarcoma: DWI and DCE-MRI parameters correlate with Ki-67 labeling index. Eur. Radiol..

[12] Dudeck O., Zeile M., Pink D., Pech M., Tunn P.U., Reichardt P., Ludwig W.D., Hamm B. (2008). Diffusion-weighted magnetic resonance imaging allows monitoring of anticancer treatment effects in patients with soft-tissue sarcomas. J. Magn. Reson. Imaging.

[13] Benz M.R., Czernin J., Allen-Auerbach M.S., Tap W.D., Dry S.M., Elashoff D., Chow K., Evilevitch V., Eckardt J.J., Phelps M.E. (2009). FDG-PET/CT imaging predicts histopathologic treatment responses after the initial cycle of neoadjuvant chemotherapy in high-grade soft-tissue sarcomas. Clin. Cancer Res..

[14] Grueneisen J., Schaarschmidt B., Demircioglu A., Chodyla M., Martin O., Bertram S., Wetter A., Bauer S., Fendler W.P., Podleska L. (2019). (18)F-FDG PET/MRI for Therapy Response Assessment of Isolated Limb Perfusion in Patients with Soft-Tissue Sarcomas. J. Nucl. Med..

[15] Theruvath A.J., Siedek F., Muehe A.M., Garcia-Diaz J., Kirchner J., Martin O., Link M.P., Spunt S., Pribnow A., Rosenberg J. (2020). Therapy Response Assessment of Pediatric Tumors with Whole-Body Diffusion-weighted MRI and FDG PET/MRI. Radiology.

[16] Grimer R.J. (2006). Size matters for sarcomas!. Ann. R. Coll. Surg. Engl..

[17] Grabellus F., Stylianou E., Umutlu L., Sheu S.Y., Lehmann N., Taeger G., Lauenstein T.C. (2012). Size-based clinical response evaluation is insufficient to assess clinical response of sarcomas treated with isolated limb perfusion with TNF-α and melphalan. Ann. Surg. Oncol..

[18] Coindre J.M., Trojani M., Contesso G., David M., Rouesse J., Bui N.B., Bodaert A., De Mascarel I., De Mascarel A., Goussot J.F. (1986). Reproducibility of a histopathologic grading system for adult soft tissue sarcoma. Cancer.

[19] Salzer-Kuntschik M., Delling G., Beron G., Sigmund R. (1983). Morphological grades of regression in osteosarcoma after polychemotherapy-study COSS 80. J. Cancer Res. Clin. Oncol..

[20] Grabellus F., Kraft C., Sheu-Grabellus S.Y., Bauer S., Podleska L.E., Lauenstein T.C., Pöttgen C., Konik M.J., Schmid K.W., Taeger G. (2011). Tumor vascularization and histopathologic regression of soft tissue sarcomas treated with isolated limb perfusion with TNF-α and melphalan. J. Surg. Oncol..

[21] Stacchiotti S., Collini P., Messina A., Morosi C., Barisella M., Bertulli R., Piovesan C., Dileo P., Torri V., Gronchi A. (2009). High-grade soft-tissue sarcomas: Tumor response assessment—Pilot study to assess the correlation between radiologic and pathologic response by using RECIST and Choi criteria. Radiology.

[22] Schuler M.K., Platzek I., Beuthien-Baumann B., Fenchel M., Ehninger G., van den Hoff J. (2015). (18)F-FDG PET/MRI for therapy response assessment in sarcoma: Comparison of PET and MR imaging results. Clin. Imaging.

[23] Cassarino G., Evangelista L., Giraudo C., Capizzi A., Carretta G., Zucchetta P., Cecchin D. (2020). 18F-FDG PET/MRI in adult sarcomas. Clin. Transl. Imaging.

[24] Leithner D., Helbich T.H., Bernard-Davila B., Marino M.A., Avendano D., Martinez D.F., Jochelson M.S., Kapetas P., Baltzer P.A.T., Haug A. (2020). Multiparametric (18)F-FDG PET/MRI of the Breast: Are There Differences in Imaging Biomarkers of Contralateral Healthy Tissue Between Patients with and without Breast Cancer?. J. Nucl. Med..

[25] Martens R.M., Koopman T., Lavini C., Ali M., Peeters C.F.W., Noij D.P., Zwezerijnen G., Marcus J.T., Vergeer M.R., Leemans C.R. (2021). Multiparametric functional MRI and (18)F-FDG-PET for survival prediction in patients with head and neck squamous cell carcinoma treated with (chemo)radiation. Eur. Radiol..

[26] Benz M.R., Allen-Auerbach M.S., Eilber F.C., Chen H.J., Dry S., Phelps M.E., Czernin J., Weber W.A. (2008). Combined assessment of metabolic and volumetric changes for assessment of tumor response in patients with soft-tissue sarcomas. J. Nucl. Med..

[27] Evilevitch V., Weber W.A., Tap W.D., Allen-Auerbach M., Chow K., Nelson S.D., Eilber F.R., Eckardt J.J., Elashoff R.M., Phelps M.E. (2008). Reduction of glucose metabolic activity is more accurate than change in size at predicting histopathologic response to neoadjuvant therapy in high-grade soft-tissue sarcomas. Clin. Cancer Res..

[28] Curnis F., Sacchi A., Corti A. (2002). Improving chemotherapeutic drug penetration in tumors by vascular targeting and barrier alteration. J. Clin. Investig..

[29] Verhoef C., de Wilt J.H., Grünhagen D.J., van Geel A.N., ten Hagen T.L., Eggermont A.M. (2007). Isolated limb perfusion with melphalan and TNF-alpha in the treatment of extremity sarcoma. Curr. Treat. Options Oncol..

[30] Lienard D., Ewalenko P., Delmotte J.J., Renard N., Lejeune F.J. (1992). High-dose recombinant tumor necrosis factor alpha in combination with interferon gamma and melphalan in isolation perfusion of the limbs for melanoma and sarcoma. J. Clin. Oncol..

[31] Eggermont A.M., Schraffordt Koops H., Klausner J.M., Kroon B.B., Schlag P.M., Liénard D., van Geel A.N., Hoekstra H.J., Meller I., Nieweg O.E. (1996). Isolated limb perfusion with tumor necrosis factor and melphalan for limb salvage in 186 patients with locally advanced soft tissue extremity sarcomas: The cumulative multicenter European experience. Ann. Surg..

[32] Eggermont A.M., Schraffordt Koops H., Liénard D., Kroon B.B., van Geel A.N., Hoekstra H.J., Lejeune F.J. (1996). Isolated limb perfusion with high-dose tumor necrosis factor-alpha in combination with interferon-gamma and melphalan for nonresectable extremity soft tissue sarcomas: A multicenter trial. J. Clin. Oncol..

[33] Brenner W., Conrad E.U., Eary J.F. (2004). FDG PET imaging for grading and prediction of outcome in chondrosarcoma patients. Eur. J. Nucl. Med. Mol. Imaging.

[34] Macpherson R.E., Pratap S., Tyrrell H., Khonsari M., Wilson S., Gibbons M., Whitwell D., Giele H., Critchley P., Cogswell L. (2018). Retrospective audit of 957 consecutive (18)F-FDG PET-CT scans compared to CT and MRI in 493 patients with different histological subtypes of bone and soft tissue sarcoma. Clin. Sarcoma Res..

[35] Eary J.F., Conrad E.U., Bruckner J.D., Folpe A., Hunt K.J., Mankoff D.A., Howlett A.T. (1998). Quantitative [F-18]fluorodeoxyglucose positron emission tomography in pretreatment and grading of sarcoma. Clin. Cancer Res..

[36] Bastiaannet E., Groen H., Jager P.L., Cobben D.C., van der Graaf W.T., Vaalburg W., Hoekstra H.J. (2004). The value of FDG-PET in the detection, grading and response to therapy of soft tissue and bone sarcomas; a systematic review and meta-analysis. Cancer Treat. Rev..

[37] Issels R., Laubender R., Lindner L., Mansmann U., Kampmann E., Verweij J., Reichardt P., Schem B., Daugaard S., Niederhagen M. (2011). Effect of FNCLCC grade 2 versus grade 3 on survival after neoadjuvant chemotherapy (NAC) plus or minus regional hyperthermia (RHT) in soft tissue sarcomas (STS): An analysis of the EORTC-ESHO Intergroup phase III study. J. Clin. Oncol..

[38] Callegaro D., Miceli R., Bonvalot S., Ferguson P., Strauss D.C., Levy A., Griffin A., Hayes A.J., Stacchiotti S., Pechoux C.L. (2016). Development and external validation of two nomograms to predict overall survival and occurrence of distant metastases in adults after surgical resection of localised soft-tissue sarcomas of the extremities: A retrospective analysis. Lancet Oncol..

[39] Fendler W.P., Lehmann M., Todica A., Herrmann K., Knösel T., Angele M.K., Dürr H.R., Rauch J., Bartenstein P., Cyran C.C. (2015). PET response criteria in solid tumors predicts progression-free survival and time to local or distant progression after chemotherapy with regional hyperthermia for soft-tissue sarcoma. J. Nucl. Med..

[40] Kubo T., Furuta T., Johan M.P., Ochi M. (2016). Prognostic significance of (18)F-FDG PET at diagnosis in patients with soft tissue sarcoma and bone sarcoma; systematic review and meta-analysis. Eur. J. Cancer.

[41] Hayashida Y., Yakushiji T., Awai K., Katahira K., Nakayama Y., Shimomura O., Kitajima M., Hirai T., Yamashita Y., Mizuta H. (2006). Monitoring therapeutic responses of primary bone tumors by diffusion-weighted image: Initial results. Eur. Radiol..

[42] Chhabra A., Ashikyan O., Slepicka C., Dettori N., Hwang H., Callan A., Sharma R.R., Xi Y. (2019). Conventional MR and diffusion-weighted imaging of musculoskeletal soft tissue malignancy: Correlation with histologic grading. Eur. Radiol..

[43] Robba T., Chianca V., Albano D., Clementi V., Piana R., Linari A., Comandone A., Regis G., Stratta M., Faletti C. (2017). Diffusion-weighted imaging for the cellularity assessment and matrix characterization of soft tissue tumour. Radiol. Med..

